# Feasibility of a pharmacy-led intervention to de-implement non-guideline-concordant proton pump inhibitor use

**DOI:** 10.1186/s43058-021-00161-6

**Published:** 2021-06-01

**Authors:** Jackson S. Musuuza, Emily Fong, Paul Lata, Katie Willenborg, Mary Jo Knobloch, Margaret J. Hoernke, Andrew R. Spiel, Jessica S. Tischendorf, Katie J. Suda, Nasia Safdar

**Affiliations:** 1 William S. Middleton Memorial Veterans Hospital, Madison, WI USA; 2 Division of Infectious Disease, Department of Medicine, University of Wisconsin School of Medicine and Public Health, University of Wisconsin, Madison, WI USA; 3 School of Pharmacy, University of Wisconsin School of Medicine and Public Health, University of Wisconsin, Madison, WI USA; 4 Division of Gastroenterology and Hepatology, Department of Medicine, University of Wisconsin School of Medicine and Public Health, University of Wisconsin, Madison, WI USA; 5 VA Pittsburgh Healthcare System, Pittsburgh, PA USA; 6 Department of Medicine, University of Pittsburgh, Pittsburgh, PA USA

## Abstract

**Background:**

Proton pump inhibitors (PPIs) are among the most prescribed medications and are often used unnecessarily. PPIs are used for the treatment of heartburn and acid-related disorders. Emerging evidence indicates that PPIs are associated with serious adverse events, such as increased risk of *Clostridioides difficile* infection. In this study, we designed and piloted a PPI de-implementation intervention among hospitalized non-intensive care unit patients.

**Methods:**

Using the Systems Engineering Initiative for Patient Safety (SEIPS) model as the framework, we developed an intervention with input from providers and patients. On a bi-weekly basis, a trainee pharmacist reviewed a random sample of eligible patients’ charts to assess if PPI prescriptions were guideline-concordant; a recommendation to de-implement non-guideline-concordant PPI therapy was sent when applicable. We used convergent parallel mixed-methods design to evaluate the feasibility and outcomes of the intervention.

**Results:**

During the study period (September 2019 to August 2020), 2171 patients with an active PPI prescription were admitted. We randomly selected 155 patient charts for review. The mean age of patients was 70.9 ± 9 years, 97.4% were male, and 35% were on PPIs for ≥5 years. The average time (minutes) needed to complete the intervention was as follows: 5 to assess if the PPI was guideline-concordant, 5 to provide patient education, and 7 to follow-up with patients post-discharge. After intervention initiation, the week-to-week mean number of PPI prescriptions decreased by 0.5 (*S*<0.0001). Barriers and facilitators spanned the 5 elements of the SEIPS model and included factors such as providers’ perception that PPIs are low priority medications and patients’ willingness to make changes to their PPI therapy if needed, respectively. Ready access to pharmacists was another frequently reported facilitator to guideline-concordant PPI. Providers recommended a PPI de-implementation intervention that is specific and tells them exactly what they need to do with a PPI treatment.

**Conclusion:**

In a busy inpatient setting, we developed a feasible way to assess PPI therapy, de-implement non-guideline-concordant PPI use, and provide follow-up to assess any unintended consequences. We documented barriers, facilitators, and provider recommendations that should be considered before implementing such an intervention on a large scale.

**Supplementary Information:**

The online version contains supplementary material available at 10.1186/s43058-021-00161-6.

Contributions to the literature

- This paper demonstrates a feasible way to assess proton pump inhibitor (PPI) therapy in an inpatient setting, to de-implement non-guideline-concordant PPI use, and to assess, and provide follow-up for unintended consequences of PPI prescription changes.
- This study provides a measurement of the time needed to complete a PPI de-implementation intervention and demonstrates a relatively short amount of time was required from pharmacists. These findings are important to pharmacy directors who are deciding if this is a feasible intervention.
- This paper provides barriers and facilitators to implementing a PPI de-implementation intervention.

## Background

Proton pump inhibitors (PPIs) are a class of medication used for the treatment of heartburn and acid-related disorders. PPIs are among the most prescribed medications in both inpatient and outpatient settings [1]. Over 61% of prescribed PPIs among patients in non-intensive care unit (ICU) settings are not guideline-concordant [2, 3]. Unnecessary and especially long-term PPI use are associated with severe adverse outcomes, such as increased incidence of *Clostridioides difficile* infection (CDI) [4–9]. In addition, overuse of PPIs is costly, with over $11 billion spent on PPIs annually in the USA [10, 11].

Unlike PPI use for stress ulcer prophylaxis (SUP) in critically ill patients, which improves clinical outcomes [12], using PPIs for SUP among non-ICU patients is not recommended [13].

De-implementation—“reducing or stopping the use or delivery of services or practices that are ineffective, unproven, harmful, overused, or inappropriate” [14, 15]—of non-guideline-concordant PPIs remains a major gap. De-implementation encounters unique challenges, as there may be more incentives to adopt a new proven innovation than to abandon a long-established practice, even when it is low value or associated with severe adverse events [15–17]. Norton and Chambers highlighted more challenges to de-implementation, including (1) loosely characterizing an intervention as evidence-based or non-evidence-based without providing details, such as effect size; (2) patient factors, such as inaccurate perceptions about the intervention; and (3) healthcare providers’ past experience of negative events and fear of medical malpractice [16].

Previous interventions to reduce unnecessary PPI use have largely been conducted in ambulatory care settings and focused on patient education about PPIs. Involvement of the full spectrum of provider stakeholders is lacking [18], and PPI overuse persists despite these previous efforts [19–21]. To address the continued need for effective interventions to promote guideline-concordant PPI use, we designed and pilot-tested a PPI de-implementation intervention and assessed its feasibility in an inpatient non-ICU setting.

## Methods

### Study design

We used a convergent parallel mixed-methods design to evaluate the intervention. Quantitative and qualitative data were collected in parallel. Quantitative methods were used to evaluate both the feasibility and outcomes of the de-implementation intervention. Qualitative methods were used to assess barriers and facilitators. We analyzed both forms of data independent of each other, but integration occurred during data interpretation [22]. We report the findings following the Template for Intervention Description and Replication (TIDieR) [23] (see Additional file 1). This study was approved by the University of Wisconsin Health Sciences Institutional Review Board.

### Participants and setting

The study was conducted from September 2019 to August 2020 at a 129-bed facility that serves ~130,000 Veterans. Adult non-ICU patients with an active PPI prescription admitted to a medical or surgical unit were included. Patients on the psychiatric, residential rehabilitation, extended stay, and hospice units were excluded because of differences in management and follow-up of these patients.

### Intervention development and content

Our intervention strategy involved developing stakeholder relationships, using evaluative and iterative strategies to define the intervention blueprint, supporting clinicians through pharmacy recommendations for PPI therapy changes, training and educating stakeholders, and engaging consumers (i.e., involving patients in the decision about their PPI therapy). This approach was based on the findings of the Expert Recommendations for Implementing Change (ERIC) study [24].

**Fig. 1:**
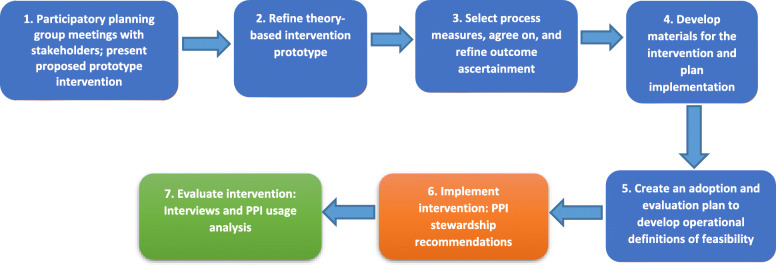
Overview of the intervention development

Intervention development involved input from pharmacists, physicians, and nurse practitioners (Fig. 1). Patients were not directly involved in developing the intervention. We started by assembling planning meetings with the antibiotic stewardship (AS) team. In our study, we wanted to leverage the existing AS program that was already performing prospective audit and feedback on antibiotic use, with the rationale that PPI de-implementation shares a common goal with AS with regard to prevention of CDI. In these meetings, we presented a prototype intervention to be refined or revamped following feedback from stakeholders, particularly inpatient pharmacist and physician workgroups. The feedback obtained guided selection of process measures, refinement of outcomes, and development of materials for the intervention. This process of intervention mapping also involved presentations to provide education and awareness about PPI effects and usage to our hospital. Two inpatient pharmacists interested in the study became its champions and supported the intervention within the facility during the implementation period. This approach of intentional collaborative efforts with stakeholders has been described as likely to improve the success of an intervention [25].

We used the Systems Engineering Initiative for Patient Safety (SEIPS) framework to design and identify barriers and facilitators to the intervention [26]. The SEIPS model describes 5 elements of a work system that can affect processes and outcomes: people, tasks, tools and technologies, organization, and environment, allowing for complete assessment of context [27]. We used the SEIPS model to consider all elements of the system that could influence non-guideline-concordant PPI prescriptions: (1) people—physicians, nurse practitioners, pharmacists, and patients; (2) tasks—evaluation of guideline-concordance; (3) tools and technologies—electronic medical record (EMR) and institutional guidelines; (4) organization—multidisciplinary research and operations team; and (5) environment—hospital units with intervention. We reasoned the SEIPS model would help us better understand how various aspects of the hospital’s system may interact to ultimately affect non-guideline-concordant PPI prescriptions, especially as factors affecting de-implementation are multi-level and cut across all elements of the work system [16].

The final pharmacy-led intervention developed over 3 months involved pharmacist review of patients’ PPI use to assess guideline-concordance [28], patient education about PPIs plus therapeutic options for their condition, and pharmacist recommendation to either stop, reduce dose, or change to a different medication class. The hospital’s Pharmacy and Therapeutics Committee approved the intervention.

### Intervention deployment

**Fig. 2:**
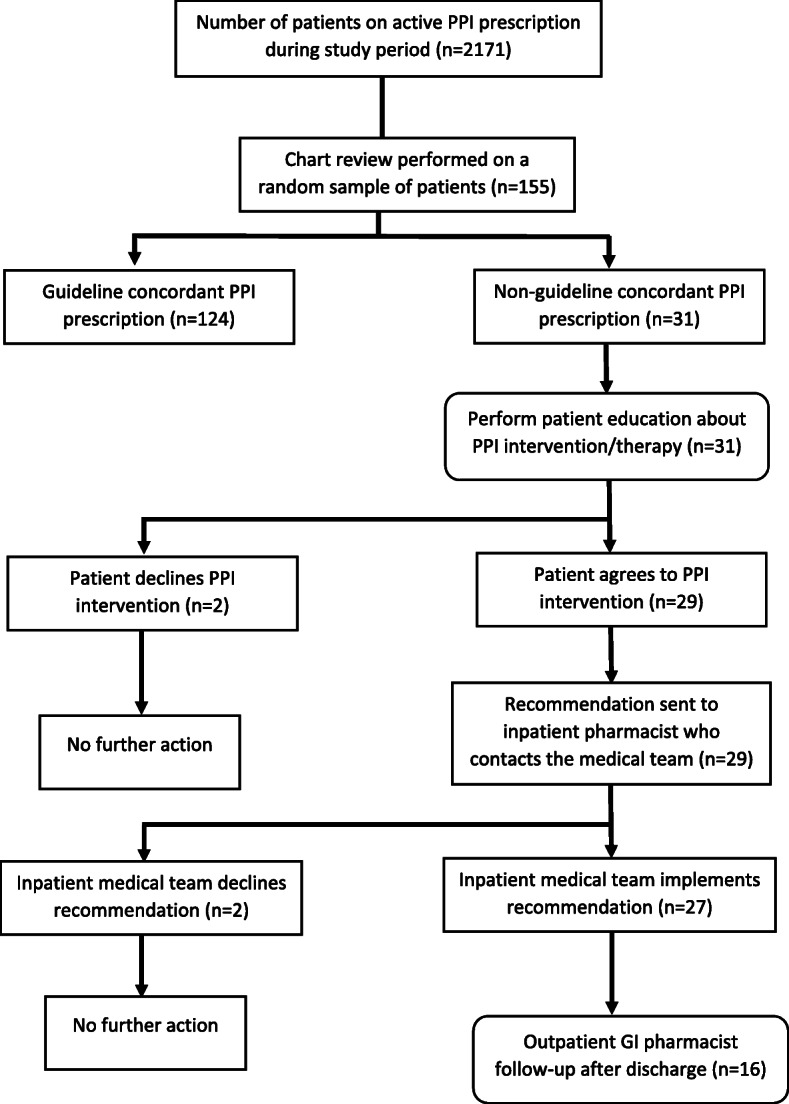
Flow chart of the intervention

On a bi-weekly basis, a list of eligible patients was generated. A trainee pharmacist reviewed a random sample of eligible patients’ charts (using patients’ unique identification numbers) to assess if the PPI prescription was guideline-concordant. (A pharmacy resident tested this process first.) A tool to decide PPI guideline-concordance was collaboratively developed through literature review and input from inpatient pharmacists, a gastroenterology physician, and the research team (see Additional file 2). If a prescription was deemed non-guideline-concordant based on indication, dose, or duration, the trainee pharmacist would provide patient education regarding the risks associated with chronic PPI use. PPI education was provided to patients individually. Patients could either accept the recommendation and provide verbal consent or could reject it without any effect on their care. If the patient consented, the trainee pharmacist would recommend de-escalation of therapy to the medical team according to a PPI de-implementation guideline developed by Farrell et al. [28]: (1) step-down PPI, (2) alternate non-PPI therapy, or (3) on-demand PPI usage by the patient. Following hospital discharge, an outpatient gastrointestinal specialty pharmacist followed up with the patient via telephone to assess tolerance and continue the taper if applicable (Fig. 2).

### Outcomes

Outcomes included acceptability and feasibility of the intervention and PPI usage (number of PPI prescriptions) before and after intervention initiation. We assessed acceptability directly from pharmacists and physicians during participatory planning meetings and through qualitative interviews. To assess the feasibility of the intervention, time to complete the intervention was recorded. This was categorized as the time to determine if the PPI prescription was guideline-concordant, time to provide patient education, and time needed to perform post-discharge follow-up.

### Quantitative data collection

We abstracted patient demographics and details related to PPI use from the EMR and entered it into a standardized form in REDCap 8.1.1® [29]. We obtained weekly PPI data for the institution for the period 8 months before and 9 months after intervention initiation.

### Qualitative data collection

In descriptive qualitative work, we documented barriers and facilitators to the intervention through qualitative interviews conducted at the end of the 6-month intervention period, and by documenting participatory planning and ongoing meetings with pharmacists and physician workgroups. We conducted phone interviews using an interview guide (see Additional file 3) with pharmacists, nurse practitioners, and physicians. Participants were identified from workgroup listservs, and we sent email invitations to participate in the interviews. Further, we used a snowball approach, where we asked the initial set of interviewees to suggest additional interviewees. The interviews were audio-recorded and transcribed. All participants provided verbal consent.

### Statistical analysis

Descriptive statistics were used for demographics, time needed to complete the intervention, and PPI usage. PPI use before and after intervention initiation was calculated using segmented regression analysis of interrupted time-series (ITS) analysis [30]. We analyzed the weekly trend of PPI prescriptions over the study period. The Huber–White sandwich estimator was used to obtain robust standard errors. We performed statistical analyses using Stata software, version 16.0 (Stata Corp. College Station, TX).

### Qualitative analysis

Content analysis was used to code the qualitative data. Two authors (JSM, MJK) independently open-coded 2 interview transcripts inductively and deductively according to the 5 elements of the SEIPS model. The authors convened, compared coding, and discussed discrepancies until they reached agreement. Once a final coding scheme was developed, one author (JSM) used it to code the remaining 8 transcripts, consulting with the other author (MJK) as necessary. Emerging themes were classified as barriers, facilitators, or recommendations for guideline-concordant PPI use. The themes were also assigned a SEIPS model element accordingly. Findings are reported according to the Standards for Reporting Qualitative Research guidelines [31].

## Results

### Patient characteristics

During the study period, 2171 patients were admitted with an active PPI prescription. We reviewed 155 randomly selected patient charts. Mean age of patients was 70.9 ± 9 years, 97.4% were male, and 35% were on PPIs for ≥ 5years; primary care providers initiated PPI therapy most frequently (Table 1). Thirty-one (20%) patients were determined to have non-guideline-concordant PPIs. The most common finding was inappropriate dose (20/31). The following recommendations were made: PPI stopped in 5 patients, PPI dose reduced in 19 patients, and PPI changed to an H2 blocker in 5 patients. All patients were educated about their condition and PPI therapy. Two recommendations were made to de-escalate PPI therapy but were declined by the inpatient pharmacist due to insight that the medical team initiated the PPIs for an alternative indication. None of the patients followed up post-discharge (*n*=16) reported unintended consequences, such as GI bleeding. The average time taken to complete the intervention was as follows: 5 min to assess if the PPI was guideline-concordant, 5 min to provide patient education prior to making changes to their PPI therapy, and 7 min to follow up with patients post-discharge.

**Table 1:** Patient characteristics and PPI usage details

Characteristic (n)	Category	n (%)
Sex (154)	Male	150 (97.4)
	Female	4 (2.6)
Race (154)	White	142 (92.2)
	Black	6 (3.9)
	Other	6 (3.9)
Duration of PPI use (156)	0–2 months	48 (30.8)
	2 months to 2 years	34 (21.8)
	3–5 years	20 (12.8)
	> 5 years	54 (34.6)
Name of current PPI (156)	Omeprazole	92 (58.9)
	Pantoprazole	58 (37.2)
	Lansoprazole	4 (2.6)
	Esomeprazole	2 (1.3)
Interval of PPI (156)	Once a day	103 (66)
	Twice a day	52 (33.3)
	As needed	1 (0.6)
Route of PPI (156)	Oral	154 (98.7)
	Intravenous	2 (1.3)
Medical provider who initiated the PPI (156)	Primary care physician	91 (58.3)
	Internal medicine	47 (30.1)
	Surgery	11 (7.1)
	Gastroenterology	7 (4.5)
Setting where the PPI was started (156)	Outpatient	98 (62.8)
	Non-ICU	53 (34)
	ICU	5 (3.2)

Mean age (standard deviation) = 70.9 years (9.3). Median length of hospital stay (interquartile range) = 11 days (13). *PPI* proton pump inhibitor, *ICU* intensive care unit

### Interrupted time series analysis

**Fig. 3:**
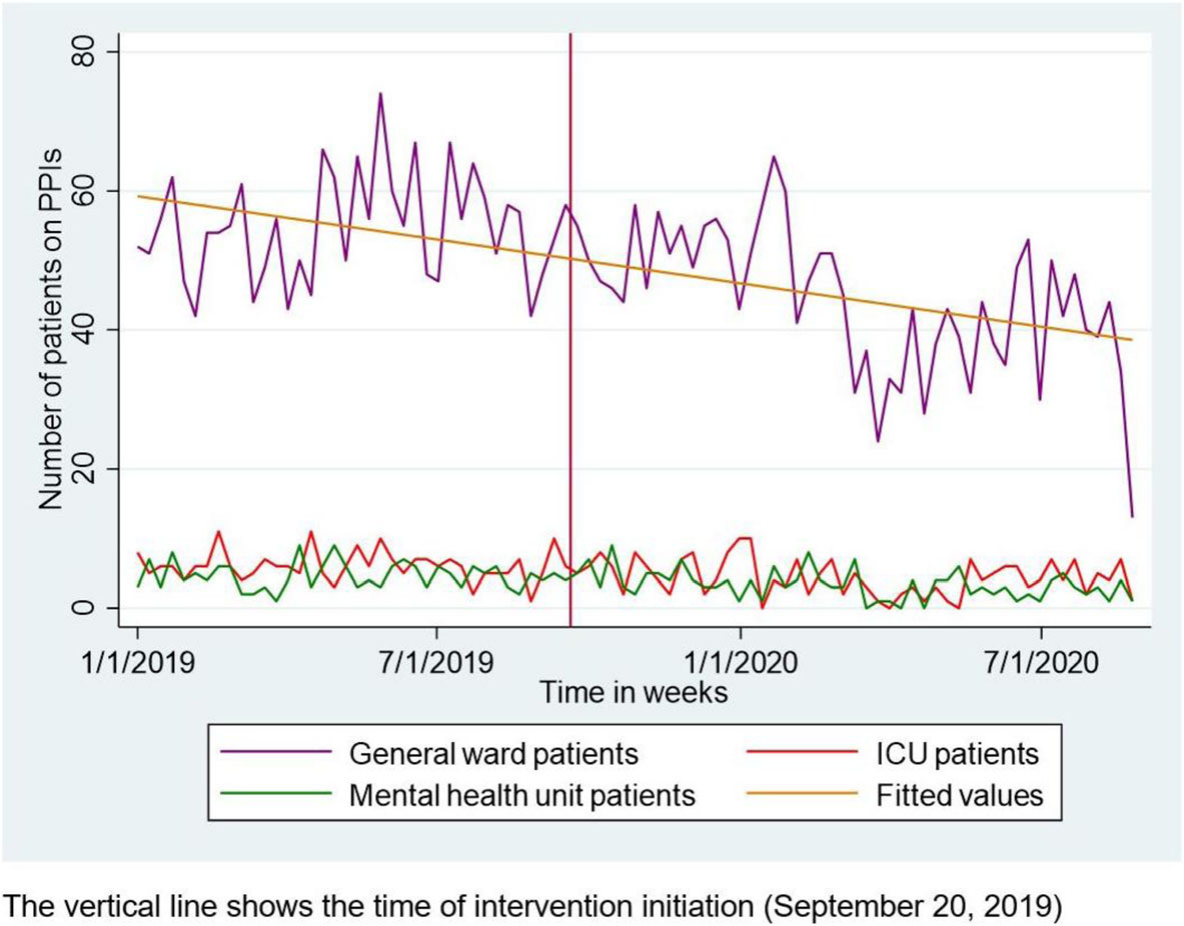
Line plot showing the number of patients on PPIs over time

Figure 3 shows the weekly number of patients on PPI prescriptions. Using regression analysis, we found a baseline mean of 53 PPI prescriptions per week in the general wards. Immediately after the intervention was implemented, the mean number of PPI prescriptions per week (or level) decreased by 2.58, but this was not statistically significant (*P*=0.411). However, after the intervention, the week-to-week (or trend) mean number of PPI prescriptions decreased by 0.5 (*P*<0.0001). The week-to-week (or trend) change in the mean number of PPI prescriptions before the intervention was 0.11 (*P*=0.273). No parameters changed significantly in non-intervention units (Table 2).

**Table 2:** Parameter estimates, standard errors, and *P*-values of segmented regression models predicting weekly numbers of PPI prescriptions over time

Parameter	Coefficient	Standard error	t-statistic	*P*-value
**General wards**				
Intercept, β_0_	52.77	2.11	25.06	<0.0001
Baseline trend, β_1_	0.11	0.09	1.10	0.273
Level change after PPI intervention, β_2_	− 2.58	3.12	− 0.83	0.411
Trend change after PPI intervention, β_3_	− 0.50	0.13	− 3.89	<0.0001
**ICU**				
Intercept, β_0_	6.58	0.65	9.96	<0.0001
Baseline trend, β_1_	− 0.02	0.03	− 0.63	0.532
Level change after PPI intervention, β_2_	− 0.29	1.04	− 0.28	0.783
Trend change after PPI intervention, β_3_	− 0.02	0.04	− 0.52	0.606

*ICU* intensive care unit, *PPI* proton pump inhibitor

### Qualitative analysis

We conducted 10 interviews total (20 min on average) with 6 physicians, 2 nurse practitioners, and 2 inpatient pharmacists. Barriers and facilitators spanned the 5 elements of SEIPS and included factors, such as providers’ perception that PPIs are low priority medications and ready access to pharmacists, respectively (Table 3). Below, we describe several salient barriers and facilitators to guideline-concordant PPI use arising from this analysis. We also identified providers’ recommendations for promoting guideline-concordant PPI use.

**Table 3:** Barriers and facilitators to guideline-concordant PPI use organized by the corresponding SEIPS element

Theme	Notes	Illustrative quotations (Q)
**Barriers to guideline-concordant PPI use**		
**Tools and technology**		
PPIs are low priority medications	Providers generally perceive PPIs as low priority medications. Even though side effects of PPIs are acknowledged, providers stated that PPIs are still considered low priority medications compared with, for example, antibiotics, where non-guideline-concordant use has far more reaching consequences.	**Q1:** The PPIs just do not sort of reach the threshold of this is important enough that I want to spend a lot of time tracking this down.
Poor awareness of ongoing intervention	Some providers were not well-informed about our ongoing PPI intervention, and some of them had not received PPI recommendations from the pharmacists.	**Q2:** I did have a pharmacist call me directly or speak with me directly, or Skype or message me directly. I think I can recall one or two occasions when there was like a brief pharmacist note put in the chart where they documented like, hey, this person is on a PPI. I reviewed the chart, and either we can de-escalate the dose or reduce the frequency. That is the most I can remember seeing is maybe one or maybe two chart interventions or documentation by the pharmacist.
**Organization**		
The GERD assumption	Providers reported that for patients whose PPI is initiated in the outpatient setting, there is a general perception that these patients have a diagnosis of GERD and should be on a PPI. For such patients, providers were reluctant to assess the appropriateness of their PPIs during admission. This results in patients taking these medications unchecked for a long duration.	**Q3:** But I think more anecdotally, without having any numbers in front of me, I think the majority probably come into the hospital already on a PPI, usually for GERD symptoms.
Hierarchy and communication	Pharmacy providers stated that they encountered instances where they recommended PPI therapy de-implementation, but the recommendation ended only with medical trainees (residents) who were not willing to make any changes to the PPI without the authorization of their seniors. This happened often on surgical wards and resulted in delay or complete inaction about the PPI if the trainee provider was not able to get timely feedback from their senior.	**Q4:** Yeah. I think some of the barriers are, usually when I call a representative of the team to discuss it, I perhaps might be talking to the surgeon who is in their first year, and they might not necessarily feel really comfortable with stopping a PPI and wanting to kind of talk to, up the chain, talk to someone else and might not necessarily be aware of the evidence and things like that.
No EMR tool dedicated to PPIs	Providers noted the absence of an EMR tool dedicated to PPIs was a barrier to guideline-concordant PPI prescription.	**Q5:** Not that I come across as an inpatient provider. I guess I would not be surprised if there were something maybe that the PCPs use, but I do not, I personally don't get any reminders or alerts or anything.
**Environment**		
Perception that chronic PPI use is an ambulatory care problem	Many providers perceive chronic PPI use an ambulatory care problem that should be handled by primary care providers (PCPs). Because of this, less effort is put towards evaluating appropriateness of PPIs for inpatients, particularly for those patients admitted while already taking a PPI.	**Q6:** A lot of the times, the impression that I get is the majority of them were already started outpatient, and they come into the hospital already on the PPIs.
Setting of PPI initiation	Providers reported that the PPIs initiated in outpatients were more of a problem than those initiated during inpatient admission. This is because providers felt they did not have sufficient details about the therapy. However, even for inpatient-initiated PPIs, there is no structured effort to ensure that they are stopped at discharge.	**Q7:** I think the bigger issue is those that are started on it inpatient, say for, whatever, stress ulcer prophylaxis, maybe those that are not necessarily on anticoagulants or on steroids, how do we make sure that they maybe get them stopped on discharge. I think that is a tougher issue.
**Person**		
Unwilling to dispute another providers decision	Providers were reluctant to discontinue a PPI if it was started in the outpatient setting as they did not want to interfere with what the treating outpatient provider had started in the context of not knowing the full patient history.	**Q8:** I think as part of the med rec, when you see that someone is on a PPI as an outpatient, we are like, well, someone, their PCP or someone thought they needed to be on a PPI, and who am I to dispute that or argue against that?
**Task**		
Time to review charts	As expected, many providers stated that time to review and find information needed to decide whether a PPI prescription is appropriate is a big challenge. Amidst many other tasks to attend to, providers find it impossible to spare time to fully evaluate a PPI prescription.	**Q9:** But it does take, it takes a lot of exploring. I know when there are pharmacy students, that's always helpful because the pharmacy student can look into some of those things a little bit more because they have more time.
**Facilitators to guideline-concordant PPI use**		
**Tools and technologies**		
Classic PPI therapy indications	Many providers reported that when the PPI was clearly indicated, they would prescribe it. This occurred in situations of classic PPI therapy indications such as high risk for gastrointestinal (GI) bleeding. However, providers do not necessarily follow up to verify the PPI gets discontinued when it is no longer indicated.	**Q10:** Or, and so it just sort of got overlooked, that maybe they should be on one, just for GI prophylaxis, and we start them because of that, because they are sort of identified as being high risk. And other times, like I said, they have an incident while they are with us that ends up requiring them, like they’ve had an active GI bleed, and then GI gets involved, and they end up being on a PPI as a result.
**Organization**		
Pharmacy residents and students able to support the intervention	The initial chart reviews to determine PPI appropriateness were carried out by a pharmacy resident or intern. Recommendations were then communicated to the inpatient pharmacist, who reached out to the patient’s treating team. Although this created some delays, it helped save the inpatient pharmacist’s time, which promoted the intervention.	**Q11:** But it does take, it takes a lot of exploring. I know when there are pharmacy students, which is always helpful because the pharmacy student can look into some of those things a little bit more because they have more time.
Ready availability of pharmacists	Providers noted that the ready availability of pharmacists at the facility and a close working relationship with them facilitated guideline-concordant PPI prescriptions. Providers would easily consult pharmacists if they needed help with medication reconciliation.	**Q12:** We work very closely with pharmacists, I would say that, I mean, if it weren't for pharmacists, we consult them for just about everything, so…
**Person**		
Patient’s willingness to make changes to their PPI medications	Through patient education about PPI therapy, we noted that patients were willing to have their PPI therapy changed if necessary. We encountered only two patients who insisted that their PPI therapy could only be changed by the outpatient provider who had initiated it.	**Q13:** If they don't have a compelling indication, it likely that at one point in time they were started on it for something like GERD or indigestion, it's usually just a conversation with the patient about trialing, reducing the dose or trialing sort of like a taper to step them down and off of it and using it as needed.
Providers’ acknowledgment of risk of PPI adverse events	Providers reported that they recognize that PPIs have adverse events and are willing to make the necessary interventions to ensure that guideline-concordant PPI prescriptions happened. However, the motivation is low, as PPIs are perceived to be low-priority medications.	**Q14:** I think we recognize that PPIs are not without their risk. I think people don't sort of look at them as a completely benign medication.
**Task**		
Acceptance of PPI recommendations by providers	Many of the providers were willing to make a PPI recommendation suggested by pharmacists. This facilitated the flow of the intervention.	**Q15:** I think out of all the options we have; I mean, I do think when pharmacists reach out to a medical team, and they say like I think we need to change this, I think most of the time, we agree with them. I think most of the time, they are right.
**Participant recommendations for promoting guideline-concordant PPI use**		
**Tools and technology**		
Forced functions in the EMR	Some providers stated that a forced function in the EMR can be an effective strategy. This would ensure that the provider thinks about the PPI before initiating it or continuing it and prevents the possibility of simply clicking through without making any changes to the PPI therapy.	**Q16:** I think people will just click through it without thinking. So, a forced function is needed
Pharmacy-driven intervention	Many providers agreed that any effective PPI intervention should be pharmacy-led, where pharmacists perform the PPI review and provide recommendations on the course of action to providers. This could be done through pharmacy notes to providers and, more importantly, through verbal communication between pharmacists and providers face-to-face, by phone, or through another platform, such as Skype.	**Q17:** I think having the pharmacists heavily involved and reviewing it and coming up with a recommendation and then potentially reaching out to the medical team at what seems to be a convenient time, like potentially later in the morning after we are rounding, I think seems good. I think even just a note, I mean just putting a note in the chart and adding the residents as additional signers.
**Organization**		
Intervene at both admission and discharge	Providers stated that an ideal PPI intervention should focus on medications the patient is taking at admission and those the patient is taking at discharge. This provides an opportunity to assess PPI prescriptions initiated in the outpatient setting and those initiated during admission.	**Q18:** I think a thorough medication review on discharge would probably be very beneficial, because I think there is a lot of protocol that happens in the hospital, and people get placed on PPIs because of protocols, especially in like ICU situations or very acute, very ill patients where it's just part of the protocols to help heal people, essentially, and they don't necessarily need them long term. And so, I think identifying, you would probably find you could stop a big percentage of PPI use from continuing in perpetuity if you med rec them on discharge as to whether or not they truly met criteria to use the medication moving forward. So, a discharge medication reconciliation is probably even more important than the admission one for appropriateness of use.
Specific intervention/recommendations	Providers mentioned that they are more likely to respond to and intervene in PPI therapy if there is a specific intervention in place. This should state, for example, how long the patient has been on a PPI and any side effects experienced, and it should clearly suggest what the provider needs to do about the PPI.	**Q19:** Well, something that, it's hard sometimes to try to track down why patients were on PPIs. So, in the future, going forward, if there is somehow a way for us to know exactly why someone was on the medicine, so it's clearly documented or clearly documented how long they should be on it, I think that would help.
Involve resident physicians	Providers recommended that involvement of resident physicians in PPI interventions is likely to increase the likelihood of the intervention happening, as residents enter most medication orders.	**Q20:** I think like if we wanted to sort of maximize the likelihood of it happening and the educational aspect of it, I think reaching out to the residents, either the intern, like the PGY1, or the more senior resident, PGY2 or PGY3, I think would be the most effective.

*PPI* proton pump inhibitor, *GERD* gastroesophageal reflux disease, *EMR* electronic medical record, *Q* illustrative quotation

#### Barrier—PPIs are low priority medications (tools and technology)

Even though side effects of PPIs are acknowledged, providers stated that PPIs are still considered low-priority medications compared to, for example, antibiotics whose non-guideline-concordant use has more far-reaching consequences (Q1).

#### Barrier—the GERD assumption (organization)

For patients whose PPI is initiated in the outpatient setting, there was a general perception that these patients have a GERD diagnosis and should be on a PPI. For such patients, providers were reluctant to assess the appropriateness of their PPI during admission. This may result in patients taking PPIs unchecked for a long duration (Q3).

#### Facilitator—ready availability of pharmacists (organization)

Providers noted that ready availability of pharmacists at the facility and a close working relationship with them facilitated guideline-concordant PPI prescriptions. Providers would easily consult pharmacists for help with medication reconciliation (Q12).

#### Facilitator—patients’ willingness to change to their PPI (person)

We noted that patients were willing to have their PPI therapy changed if necessary. We encountered only 2 patients who insisted that their PPI therapy could only be changed by the outpatient provider who had initiated it (Q13).

## Discussion

We designed an intervention to de-implement non-guideline-concordant PPI use in the inpatient setting that was feasible and decreased the week-to-week mean number of PPI prescriptions. We found that providers’ perception that PPIs are low priority medications was a major barrier, while ready access to pharmacists was a major facilitator to guideline-concordant PPI use.

Our study builds on previous work to address the problem of non-guideline-concordant PPI use. Some prior studies were conducted in the outpatient setting and may not address factors unique to the inpatient setting, the focus of our study [32, 33]. However, a study by Michal et al. in adult non-ICU hospitalized patients found pharmacists’ review of PPIs and recommendation to physicians led to a 25% decrease in PPI prescriptions [34]. Unlike our study in which the intervention lasted for 9 months, the intervention period in Michal et al. was only 1 month. Sustainability of interventions is more likely with a long intervention period [35]. In addition, Gleason et al. found that pharmacists take on average 21.2 min to conduct a complete medicine reconciliation intervention [36]. Our intervention took a total of 10 min, a comparatively short time.

Our pilot intervention was feasible and reduced week-to-week PPI prescriptions following the intervention, without reported unintended adverse effects of PPI de-implementation. The latter, rather than an immediate decline in PPI use, is consistent with the evidence that successful de-implementation efforts require regular messaging after the initial rollout of the intervention [15]. Other than the ongoing medicine reconciliation efforts at our facility, there were no other new medication-related interventions during the intervention period. Moreover, no changes in PPI prescriptions were observed in the non-intervention units (ICU and mental health unit). Although there was a downtrend in PPI prescriptions prior to the intervention (Fig. 3), this trend was not statistically significant, unlike the significant week-to-week decline in PPI prescriptions observed after the intervention. Given that this was a single-site study, it is difficult to ascertain the clinical significance of this week-to-week decline. Larger multisite studies are needed to further assess the effect of such a PPI de-implementation intervention on the number of PPI prescriptions and patient outcomes.

We noted barriers to guideline-concordant PPI use that spanned all elements of the SEIPS model. First, providers perceived PPIs as low priority medications. This may be due in part because most PPI adverse events take a long time to manifest [37, 38]. Hence, there is a need for more education and provision of evidence of serious adverse events of PPIs to providers. Evidence is one of the major driving forces of change in the process of de-implementation [17]. A second barrier was the “GERD assumption,” an organizational barrier where inpatient providers have a general perception that patients who are hospitalized while on a PPI therapy have a diagnosis of GERD. For such patients, inpatient providers were reluctant to intervene in PPI therapy. Mitigating this barrier may require targeted provider education.

Similarly, the facilitators spanned all 5 SEIPS model elements. Prescribing providers noted that ready access to a pharmacist was paramount in ensuring guideline-concordant PPI use. Notably, patients were willing to make changes to their PPI treatment if needed. This is important because providers can be assured of patient engagement in the process, which improves adherence to the proposed medication changes [39].

Providers consistently recommended that they would like a specific intervention that directs them on what to do for the patient. This is important for saving time, one of the barriers stated by providers. In addition, providers recommended a pharmacy-driven intervention. This would fit well in the *medication reconciliation* role that pharmacists are already performing, albeit with special emphasis on PPIs. Medication reconciliation, the “process of comparing a patient's medication orders to all of the medications that the patient has been taking,” is done to prevent errors, such as duplication, dosing errors, and duration errors, which can potentially lead to patient harm. One strategy used is pharmacy-led review of medications at transition points, such as from ICU to ward or at discharge. Instituting PPI review at such times may be a plausible approach to reducing non-guideline-concordant PPI use [40–42].

Strengths of our study include the involvement of patients through education prior to changes in their PPI therapy. Patient involvement is associated with adherence to interventions [39]. Another strength was that the development of our PPI de-implementation intervention was informed by provider input, where we started by assembling planning meetings with an already existing AS team. However, any organized inpatient team, such as pharmacy and therapeutics committees, can be a starting point. Limitations included the single-site nature of the study. However, the use of the SEIPS model, which captures contextual, system-wide factors, increases the applicability of our results.

## Conclusions

We developed an approach to assess PPI therapy, de-implement non-guideline-concordant PPI use, and provide follow-up to assess unintended consequences of PPI modification in an inpatient setting. Providers’ perceptions that PPIs are low priority medications was a frequently reported barrier, while ready access to pharmacists was a frequently reported facilitator to guideline-concordant PPI use. Future studies should consider provider recommendations, including using a provider-friendly intervention, forced EMR functions, and pharmacy-driven interventions.

## Supplementary Information

**Additional file 1.** The TIDieR (Template for Intervention Description and Replication) Checklist

**Additional file 2: Table 1.** Proton pump inhibitor indications, doses, and durations of use

**Additional file 3.** PPI usage in the VA—interview guide for clinicians

## Data Availability

The data on which conclusions are based is available from authors upon request.

## Abbreviations

PPI: Proton pump inhibitors
EMR: Electronic medical records
ICU: Intensive care unit
SEIPS: Systems Engineering Initiative for Patient Safety
CDI: *Clostridioides difficile* infection
GERD: Gastroesophageal reflux disease

## References

[1] Nehra AK, Alexander JA, Loftus CG, Nehra V (2018). In Reply-A relationship between proton pump inhibitors and hypomagnesemia?. Mayo Clin Proc..

[2] Haroon M, Yasin F, Gardezi SK, Adeeb F, Walker F (2013). Inappropriate use of proton pump inhibitors among medical inpatients: a questionnaire-based observational study. JRSM Short Rep..

[3] Eid SM, Boueiz A, Paranji S, Mativo C, Landis R, Abougergi MS (2010). Patterns and predictors of proton pump inhibitor overuse among academic and non-academic hospitalists. Intern Med..

[4] Kwok CS, Arthur AK, Anibueze CI, Singh S, Cavallazzi R, Loke YK (2012). Risk of Clostridium difficile infection with acid suppressing drugs and antibiotics: meta-analysis. Am J Gastroenterol..

[5] Eom CS, Jeon CY, Lim JW, Cho EG, Park SM, Lee KS (2011). Use of acid-suppressive drugs and risk of pneumonia: a systematic review and meta-analysis. CMAJ..

[6] Filion KB, Chateau D, Targownik LE, Gershon A, Durand M, Tamim H, Teare GF, Ravani P, Ernst P, Dormuth CR, the CNODES Investigators (2014). Proton pump inhibitors and the risk of hospitalisation for community-acquired pneumonia: replicated cohort studies with meta-analysis. Gut..

[7] Lazarus B, Chen Y, Wilson FP, Sang Y, Chang AR, Coresh J, Grams ME (2016). Proton Pump Inhibitor Use and the Risk of Chronic Kidney Disease. JAMA Intern Med..

[8] Zhou B, Huang Y, Li H, Sun W, Liu J (2016). Proton-pump inhibitors and risk of fractures: an update meta-analysis. Osteoporos Int..

[9] Cheungpasitporn W, Thongprayoon C, Kittanamongkolchai W, Srivali N, Edmonds PJ, Ungprasert P, O'Corragain OA, Korpaisarn S, Erickson SB (2015). Proton pump inhibitors linked to hypomagnesemia: a systematic review and meta-analysis of observational studies. Ren Fail..

[10] Heidelbaugh JJ, Goldberg KL, Inadomi JM (2009). Overutilization of proton pump inhibitors: a review of cost-effectiveness and risk in PPI. Am J Gastroenterol ..

[11] Heidelbaugh JJ, Kim AH, Chang R, Walker PC (2012). Overutilization of proton-pump inhibitors: what the clinician needs to know. Therap Adv Gastroenterol..

[12] Alshamsi F, Belley-Cote E, Cook D, Almenawer SA, Alqahtani Z, Perri D, Thabane L, al-Omari A, Lewis K, Guyatt G, Alhazzani W (2016). Efficacy and safety of proton pump inhibitors for stress ulcer prophylaxis in critically ill patients: a systematic review and meta-analysis of randomized trials. Crit Care..

[13] Qadeer MA, Richter JE, Brotman DJ (2006). Hospital-acquired gastrointestinal bleeding outside the critical care unit: risk factors, role of acid suppression, and endoscopy findings. J Hosp Med..

[14] Norton WE, Kennedy AE, Chambers DA (2017). Studying de-implementation in health: an analysis of funded research grants. Implement Sci.

[15] Prusaczyk B, Swindle T, Curran G (2020). Defining and conceptualizing outcomes for de-implementation: key distinctions from implementation outcomes. Implement Sci Commun..

[16] Norton WE, Chambers DA (2020). Unpacking the complexities of de-implementing inappropriate health interventions. Implementation Sci.

[17] Powers BW, Jain SH, Shrank WH (2020). De-adopting low-value care: evidence, eminence, and economics. JAMA..

[18] Haastrup P, Paulsen MS, Begtrup LM, Hansen JM, Jarbøl DE (2014). Strategies for discontinuation of proton pump inhibitors: a systematic review. Fam Pract..

[19] Wahking RA, Steele RL, Hanners RE, Lockwood SM, Davis KW (2018). Outcomes from a pharmacist-led proton pump inhibitor stewardship program at a single institution. Hosp Pharm..

[20] Reeve E, Andrews JM, Wiese MD, Hendrix I, Roberts MS, Shakib S (2015). Feasibility of a patient-centered deprescribing process to reduce inappropriate use of proton pump inhibitors. Ann Pharmacother.

[21] Jaynes M, Kumar AB (2019). The risks of long-term use of proton pump inhibitors: a critical review. Ther Adv Drug Saf..

[22] Fetters MD, Curry LA, Creswell JW (2013). Achieving integration in mixed methods designs-principles and practices. Health Serv Res..

[23] Hoffmann TC, Glasziou PP, Boutron I, Milne R, Perera R, Moher D, Altman DG, Barbour V, Macdonald H, Johnston M, Lamb SE, Dixon-Woods M, McCulloch P, Wyatt JC, Chan AW, Michie S (2014). Better reporting of interventions: template for intervention description and replication (TIDieR) checklist and guide. BMJ..

[24] Waltz TJ, Powell BJ, Matthieu MM, Damschroder LJ, Chinman MJ, Smith JL, Proctor EK, Kirchner JAE (2015). Use of concept mapping to characterize relationships among implementation strategies and assess their feasibility and importance: results from the Expert Recommendations for Implementing Change (ERIC) study. Implement Sci..

[25] Huynh TM (2013). Innovators and early adopters of population health in healthcare: real and present opportunities for healthcare-public health collaboration. Healthc Pap..

[26] Holden RJ, Carayon P, Gurses AP, Hoonakker P, Hundt AS, Ozok AA, Rivera-Rodriguez AJ (2013). SEIPS 2.0: a human factors framework for studying and improving the work of healthcare professionals and patients. Ergonomics..

[27] Shekelle PG, Pronovost PJ, Wachter RM, Taylor SL, Dy SM, Foy R, et al. Advancing the science of patient safety. Ann Intern Med. 154(10):693–6. 10.7326/0003-4819-154-10-201105170-00011 21576538

[28] Farrell B, Pottie K, Thompson W, Boghossian T, Pizzola L, Rashid FJ, Rojas-Fernandez C, Walsh K, Welch V, Moayyedi P (2017). Deprescribing proton pump inhibitors: evidence-based clinical practice guideline. Can Fam Physician..

[29] Harris PA, Taylor R, Minor BL, Elliott V, Fernandez M, O'Neal L (2019). The REDCap consortium: Building an international community of software platform partners. J Biomed Inform.

[30] Wagner AK, Soumerai SB, Zhang F, Ross-Degnan D (2002). Segmented regression analysis of interrupted time series studies in medication use research. J Clin Pharm Ther..

[31] O'Brien BC, Harris IB, Beckman TJ, Reed DA, Cook DA (2014). Standards for reporting qualitative research: a synthesis of recommendations. Acad Med.

[32] Inadomi JM, Jamal R, Murata GH, Hoffman RM, Lavezo LA, Vigil JM, Swanson KM, Sonnenberg A (2001). Step-down management of gastroesophageal reflux disease. Gastroenterology..

[33] Zwisler JE, Jarbøl DE, Lassen AT, Kragstrup J, Thorsgaard N, Schaffalitzky de Muckadell OB (2015). Placebo-controlled discontinuation of long-term acid-suppressant therapy: a randomised trial in general practice. Int J Family Med.

[34] Michal J, Henry T, Street C (2016). Impact of a pharmacist-driven protocol to decrease proton pump inhibitor use in non-intensive care hospitalized adults. Am J Health Syst Pharm..

[35] Walugembe DR, Sibbald S, Le Ber MJ, Kothari A (2019). Sustainability of public health interventions: where are the gaps?. Health Res Policy Syst..

[36] Gleason KM, McDaniel MR, Feinglass J, Baker DW, Lindquist L, Liss D (2010). Results of the Medications at Transitions and Clinical Handoffs (MATCH) study: an analysis of medication reconciliation errors and risk factors at hospital admission. J Gen Intern Med..

[37] Lyon J (2017). Study questions use of acid suppressors to curb mild infant reflux. JAMA..

[38] Strand DS, Kim D, Peura DA (2017). 25 years of proton pump inhibitors: a comprehensive review. Gut Liver..

[39] Sharma AE, Rivadeneira NA, Barr-Walker J, Stern RJ, Johnson AK, Sarkar U (2018). Patient engagement in health care safety: an overview of mixed-quality evidence. Health Aff (Project Hope)..

[40] Eggink RN, Lenderink AW, Widdershoven JW, van den Bemt PM (2010). The effect of a clinical pharmacist discharge service on medication discrepancies in patients with heart failure. Pharm World Sci.

[41] Mekonnen AB, McLachlan AJ, Brien JA (2016). Effectiveness of pharmacist-led medication reconciliation programmes on clinical outcomes at hospital transitions: a systematic review and meta-analysis. BMJ Open..

[42] Almanasreh E, Moles R, Chen TF (2016). The medication reconciliation process and classification of discrepancies: a systematic review. Br J Clin Pharmacol..

