# Supplementary material for: Feasibility of a pharmacy-led intervention to de-implement non-guideline-concordant proton pump inhibitor use
Source: Implement Sci Commun. 2021 Jun 1;2:59. doi: 10.1186/s43058-021-00161-6 (PMC8171048; doi:10.1186/s43058-021-00161-6)
Supplement: Supplementary file 2 — Additional file 2: Table 1. Proton pump inhibitor indications, doses, and durations of use [file 43058_2021_161_MOESM2_ESM.pdf]

Additional file 2.Table 1. Proton pump inhibitor indications, doses, and durations of use.

Condition	Dose	Duration	Notes
Achalasia after myotomy	For symptom control	Indefinitely	
Sleeve gastrectomy esophagectomy	Symptom control	Indefinitely	
Barrett's Esophagus	Daily (maybe BID in refractory symptoms)	Indefinitely	Some require BID for symptom control
Dyspepsia (chronic epigastric pain for greater than 1 month)	Daily	PRN	Attempt withdrawal every 6-12 months
Eosinophilic esophagitis	Daily or BID	Indefinitely	
Endoscopic dilation of stricture or ring	Daily or BID	Indefinitely	
Heartburn/GERD	Daily	8 weeks - indefinite	Lowest effective dose
Erosive esophagitis	Daily or BID	8 weeks – indefinite	
Peptic Ulcer disease	Daily or BID	4-8 weeks	Duration depends on etiology
H. Pylori	Daily or BID	14 days	If Ulcer – 8 weeks
Portal Hypertensive gastropathy	Controversial		
History of NSAID bleeding ulcer in patient requiring ongoing NSAID	Daily	For duration requiring NSAID	Cox-2 preferred
History of low dose ASA bleeding ulcer requiring ongoing aspirin for secondary ASCVD prevention	Daily	For duration requiring aspirin	
Antiplatelet therapy with history of previous complicated ulcer	Daily	For duration requiring antiplatelet therapy	
Antiplatelet or anticoagulant or NSAID with 3 or more risk factors (≥65 years (or 70?) old, hx ulcer, high dose NSAID, concurrent ASA, corticosteroids, anticoagulant use, alcohol use?), OR history of upper GI bleed	Daily	For duration that meets criteria	

Lung transplant	Daily	Indefinitely	To prevent chronic rejection
All other transplant patients	Daily	Varies	Consult transplant RPH
Post LVAD	Daily	Indefinite	To prevent LVAD induced gastrointestinal bleed, although only ~25% of GIB related to LVAD are ulcer mediated (most are due to small intestinal AVM)
Post atrial fibrillation ablation	Daily	30 days	To prevent esophageal damage (erosion, ulceration, or perforation) leading to atrio-esophageal fistula

Note. LVAD: left ventricular assist device; AVM: arteriovenous malformation; GERD: gastroesophageal reflux disease; ASCVD: Atherosclerotic Cardiovascular Disease; NSAID: Nonsteroidal anti-inflammatory drugs; ASA: Aspirin; BID: twice a day; PRN: as needed.
