# Supplementary material for: Feasibility of a pharmacy-led intervention to de-implement non-guideline-concordant proton pump inhibitor use
Source: Implement Sci Commun. 2021 Jun 1;2:59. doi: 10.1186/s43058-021-00161-6 (PMC8171048; doi:10.1186/s43058-021-00161-6)
Supplement: Supplementary file 3 — Additional file 3. PPI usage in the VA—interview guide for clinicians [file 43058_2021_161_MOESM3_ESM.pdf]

PPI USAGE IN THE VA—INTERVIEW GUIDE FOR CLINICIANS

Date: ____________________ Begin time: _________________ End time: __________________

Introduction:

Thank you for taking the time to talk to me. In this interview, we will discuss your perceptions about the use of proton pump inhibitors (PPIs). The information obtained will help us capture lessons that can be used in future interventions.

The interview will take about 20 minutes. Your responses to all questions will be kept anonymous and confidential. If you inadvertently share any confidential information about yourself or patients, that information will be deleted from the recording and any documents created. Any information we include in write-ups or publications will not identify you as the respondent.

To ensure I do not miss any of your comments, I would like to audio record our conversation. Is that okay? (*If yes, turn on two recorders.)*

Do you have any questions about what I have just explained?

Are you still willing to participate in this interview?

Topic Area I: Background and contextual issues

- What is your title/position?
- How long have you been at this institution?
- Please describe to me the responsibilities your position entails. Or: What tasks do you perform on a day-to-day basis?

Topic Area II: Perceptions of PPI usage

Let us focus our discussion now and talk specifically about gastric acid suppression at the Madison VA hospital inpatient units.

In your perception, what is the current culture regarding use PPIs on the teams you work with? (SEIPS - organization)

For example:

- When are PPIs ordered?
- For what conditions are PPIs prescribed?
- What are the common agents used?

- In your perception, what is the relative frequency with which these agents are used?

How do you feel about the current culture in the unit(s) or teams you work regarding PPI use?

- How much of a problem do you think is inappropriate use, either under- or over-prescription, of PPIs is in the unit (s) you work at?

What opinions do you have about the different roles in this unit surrounding initiation of PPIs?

For example:

- What role do pharmacists play?
- What role do providers (physicians/NPs) play? (**SEIPS – person)**

Topic Area III: Current Practices

What is your current practice for prescribing a PPI to patients admitted to the unit/to the team you work with?

- What informs your decision to start a PPI?
- What tools or resources do you consult to inform your decisions regarding PPI use? **(SEIPS - technology and tools)**
- What processes are in place to facilitate ordering of a PPI? **(SEIPS - technology and tools)**

Further question, if needed:

- Describe any "reminders" built in to your workflow - either electronic or otherwise - that facilitate ordering of a PPI. **(SEIPS - technology and tools)**

How do you make decisions regarding the specific PPI medication you prescribe? (**SEIPS – task)**

Further questions:

- Which agent do you prefer? Why this preference?
- If you prescribe other agents, with what relative frequency?

If a patient is on a PPI at the time of admission, what is your practice in reconciling this medication? (**SEIPS – task)**

Further questions:

- In which scenarios, if any, would you consider discontinuing a PPI at admission?
- In which scenarios, if any, would you consider changing to a different class of gastric acid suppression medication such as an H2 blocker?

How do you make the decision to discontinue a PPI therapy? **(SEIPS - task)**

Further questions:

- What processes are in place to facilitate discontinuation of a PPI? **(SEIPS - technology and tools)**
- Tell me about conversations you have had regarding discontinuation of a PPI? **(SEIPS - person)**

Describe initiatives you may recall in the past to attempt to reduce the prescribing of PPIs? (**SEIPS - organization**)

There is an ongoing pharmacy-led inpatient effort to reduce prescribing of PPIs, how have you interacted with this effort at all?

- For example, have received any requests to reduce dose, stop a PPI or change to an H2 receptor blocker? (**SEIPS - organization**)

What are your recommendations for an ideal intervention to reduce PPI prescribing? **(SEIPS - technology and tools)**

If you were to design a PPI-implementation intervention, how would it be?

- What challenges would you anticipate?
- What would are some of the resources you think would make the intervention successful?
- What kind of people or committees would you bring on-board?
- How would you involve the EHR/CPRS if any?
- If tapering is an option, how should this be approached?

Upon PPI deprescribing (stopping, changing dose or changing to another class):

- What monitoring needs to be done? How often?
- How should symptoms be managed?

What other approaches can help with PPI deprescribing (reducing PPI prescription)?

Additional questions:

Tell me about discussions you have had with patients regarding the use of PPIs. **(SEIPS - person)**

You may perceive based on our discussion, or your own experience, that there is over prescription of PPIs at our facility.

- What barriers may exist to optimizing PPI use?
- What can we do to change practice?

What is your perception of how leadership feels about PPI overuse? Specifically for inpatients? (SEIPS - organization)

What opportunities exist for leadership and staff to discuss ideas around evidence based use of acid suppressing medications?

- If a reasonable change in practice is proposed, who is involved in promoting this change?
- Does the leadership support these changes and promote them? (**SEIPS - organization**)

How different do you think PPI use is different in the ICU compared to non-ICU inpatient units?

In the ICU, sometimes PPIs are used for stress ulcer prophylaxis **(SEIPS - organization)**

- When is stress ulcer prophylaxis ordered?
- What agents are used for stress ulcer prophylaxis?
- In your perception, what is the relative frequency with which these agents are used?

How different do you think PPI use is different in the ICU compared to non-ICU inpatient units?

In the ICU, sometimes PPIs are used for stress ulcer prophylaxis **(SEIPS - organization)**

- When is stress ulcer prophylaxis ordered?
- What agents are used for stress ulcer prophylaxis?
- In your perception, what is the relative frequency with which these agents are used?

Thank you for your time.

PPI USAGE IN THE VA—INTERVIEW GUIDE FOR PHARMACISTS

Date: ____________________ Begin time: _________________ End time: __________________

Introduction:

Thank you for taking the time to talk to me. In this interview, we will discuss your perceptions about the use of proton pump inhibitors (PPIs). The information obtained will help us capture lessons that can be used in future interventions.

The interview will take about 20 minutes. Your responses to all questions will be kept anonymous and confidential. If you inadvertently share any confidential information about yourself or patients, that information will be deleted from the recording and any documents created. Any information we include in write-ups or publications will not identify you as the respondent.

To ensure I do not miss any of your comments, I would like to audio record our conversation. Is that okay? (*If yes, turn on two recorders.)*

Do you have any questions about what I have just explained?

Are you still willing to participate in this interview?

Topic Area I: Background and contextual issues

- What is your title/position?
- How long have you been at this institution?
- Please describe to me the responsibilities your position entails. Or: What tasks do you perform on a day-to-day basis?

Topic Area II: Perceptions of PPI usage
Let us focus our discussion now and talk specifically about gastric acid suppression at the Madison VA hospital inpatient units.

In your perception, what is the current culture regarding use PPIs on the teams you work with? (SEIPS - organization)

For example:

- When are PPIs ordered?
- For what conditions are PPIs prescribed?
- What are the common agents used?
- In your perception, what is the relative frequency with which these agents are used?

How do you feel about the current culture on the team you work regarding PPI use?

- How much of a problem do you think is inappropriate use, either under- or over-prescription, of PPIs is in the team you work with?

- What barriers to optimal PPI use may exist at our facility?

- What can we do to change practice?

What opinions do you have about the different roles in your team surrounding initiation of PPIs?
For example:

- What role do pharmacists play?

- What role do physicians or other providers play? (**SEIPS – person)**

Topic Area III: Current Practices

What is the current practice for prescribing a PPI to patients admitted to your team?

- What tools or resources do you use to inform your decisions regarding PPI use for your patients? **(SEIPS - technology and tools)**

- Are there processes are in place to facilitate ordering of a PPI? **(SEIPS - technology and tools)**

From your experience and practice, which agent do you prefer? Why this preference?

If a patient is on a PPI at the time of admission, what is your practice in reconciling this medication? (**SEIPS – task)**

Further questions

- In which scenarios, if any, would you consider discontinuing this medication on admission to your team or unit?

- In which scenarios, if any, would you consider changing to a different class of gastric acid suppression medication such as an H2 blocker?

- How do you make the decision to discontinue or change a PPI therapy to another class? **(SEIPS - task)**
    - Do you consult the treating physician before making the decisions above?

    - Tell me about this process

Further questions:

- What processes are in place to facilitate discontinuation of a PPI? (SEIPS - technology and tools)

- Tell me about conversations you have had with your team regarding discontinuation of a PPI? (SEIPS - person)

- Tell me about the provider variability you have encountered about use of PPIs on the unit (s) or teams you work with? (SEIPS - person**)**

- Tell me about discussions you have had with patients regarding the use of PPIs. **(SEIPS - person)**

Describe initiatives you may recall in the past to attempt to reduce the prescribing of PPIs? (**SEIPS - organization**)

There is an ongoing pharmacy-led inpatient effort to reduce prescribing of PPIs, how have you interacted with this effort at all?

- For example, have received any requests to reduce dose, stop a PPI or change to an H2 receptor blocker? (**SEIPS - organization**)

What are your recommendations for an ideal intervention to reduce PPI prescribing? **(SEIPS - technology and tools)**

If you were to design a PPI-implementation intervention, how would it be?

- What challenges would you anticipate?
- What would are some of the resources you think would make the intervention successful?
- What kind of people or committees would you bring on-board?
- How would you involve the EHR/CPRS if any?
- If tapering is an option, how should this be approached?

Upon PPI deprescribing (stopping, changing dose or changing to another class):

- What monitoring needs to be done? How often?
- How should symptoms be managed?

What other approaches can help with PPI deprescribing (reducing PPI prescription)?

<u>Additional questions:</u>

What is your perception of how leadership feels about PPI overuse? Specifically for inpatients? (**SEIPS - organization**)

What opportunities exist for leadership and staff to discuss ideas around evidence based use of acid suppressing medications?

- If a reasonable change in practice is proposed, who is involved in promoting this change?
- Does the leadership support these changes and promote them? (**SEIPS - organization**)

How different do you think PPI use is different in the ICU compared to non-ICU inpatient units?

Describe initiatives you may recall in the past to attempt to reduce the prescribing of PPIs? (SEIPS - organization)

Thank you for your time.
